# ICoVeR – an interactive visualization tool for verification and refinement of metagenomic bins

**DOI:** 10.1186/s12859-017-1653-5

**Published:** 2017-05-02

**Authors:** Bertjan Broeksema, Magdalena Calusinska, Fintan McGee, Klaas Winter, Francesco Bongiovanni, Xavier Goux, Paul Wilmes, Philippe Delfosse, Mohammad Ghoniem

**Affiliations:** 1 Environmental Research and Innovation Department, Luxembourg Institute of Science and Technology, 41 rue du Brill, L-4422 Belvaux, Luxembourg; 2 Johann Bernoulli Institute for Mathematics and Computer Science, University of Groningen, 9747 AG Groningen, The Netherlands; 3 Luxembourg Centre for Systems Biomedicine, University of Luxembourg, 7 avenue des Hauts-Fourneaux, L-4362 Esch-sur-Alzette, Luxembourg

**Keywords:** Contig bin visualization, Genome reconstruction, Metagenomics, Software

## Abstract

**Background:**

Recent advances in high-throughput sequencing allow for much deeper exploitation of natural and engineered microbial communities, and to unravel so-called “microbial dark matter” (microbes that until now have evaded cultivation). Metagenomic analyses result in a large number of genomic fragments (contigs) that need to be grouped (binned) in order to reconstruct draft microbial genomes. While several contig binning algorithms have been developed in the past 2 years, they often lack consensus. Furthermore, these software tools typically lack a provision for the visualization of data and bin characteristics.

**Results:**

We present ICoVeR, the Interactive Contig-bin Verification and Refinement tool, which allows the visualization of genome bins. More specifically, ICoVeR allows curation of bin assignments based on multiple binning algorithms. Its visualization window is composed of two connected and interactive main views, including a parallel coordinates view and a dimensionality reduction plot. To demonstrate ICoVeR’s utility, we used it to refine disparate genome bins automatically generated using MetaBAT, CONCOCT and MyCC for an anaerobic digestion metagenomic (AD microbiome) dataset. Out of 31 refined genome bins, 23 were characterized with higher completeness and lower contamination in comparison to their respective, automatically generated, genome bins. Additionally, to benchmark ICoVeR against a previously validated dataset, we used Sharon’s dataset representing an infant gut metagenome.

**Conclusions:**

ICoVeR is an open source software package that allows curation of disparate genome bins generated with automatic binning algorithms. It is freely available under the GPLv3 license at https://git.list.lu/eScience/ICoVeR. The data management and analytical functions of ICoVeR are implemented in R, therefore the software can be easily installed on any system for which R is available. Installation and usage guide together with the example files ready to be visualized are also provided via the project wiki. ICoVeR running instance preloaded with AD microbiome and Sharon’s datasets can be accessed via the website.

**Electronic supplementary material:**

The online version of this article (doi:10.1186/s12859-017-1653-5) contains supplementary material, which is available to authorized users.

## Background

Rapid improvement of high-throughput sequencing technologies allows for much deeper exploitation of microbial communities, including bacteria, archaea and microeukaryotes. While only a small fraction of the microbial phylogenetic diversity is represented by cultivated organisms [1], metagenomics (shotgun DNA sequencing) allows reconstruction of microbial genomes (partial and/or complete) directly from environmental samples without cultivation. However, the binning of assembled metagenomic contigs into individual genomes still remains a significant challenge.

The combination of tetra-nucleotide frequencies (TNFs; sequence-dependent contig binning) with contigs’ differential abundance spectra (sequence-independent contig binning) has resulted recently in the development of multiple (fully) automated contig binning approaches [2–6]. Even though fully automated genome binning allows the processing of large amounts of sequencing data, the different binning algorithms often result in redundant or overlapping genome bins. Moreover, depending on the chosen parameters, even an individual binning algorithm may provide different binning results. This makes it difficult for a user to determine which configuration produces the best results. Alternatively, one might want to combine bin assignments from different tools (or parameter configurations) in order to get the “consensus” set of bins, similarly to a recently proposed merged assembly concept [7].

Therefore, further verification of bin completeness and contamination (based on the presence of essential single copy genes, ESCGs) as well as subsequent bin refinement are necessary. The first task has been recently addressed by the development of CheckM [8], an automated tool that estimates the completeness and contamination of draft genomes (population genomes recovered from metagenomic data) using a set of marker genes that are specific to a genome’s inferred lineage. The second issue concerning the refinement of genomic bins would ideally require an interactive framework allowing for visualizing several automated binning outputs in order to perform further supervised binning. To our knowledge, anvi’o [9] is the only interactive tool that enables for such visualization and for subsequent human-assisted improvement of automatically generated genome bins resulting from multiple binning algorithms. Even though, the anvi’o metagenomic workflow enables the user to interactively work with the data and to perform a supervised binning using a *real-time* display of bin completeness and contamination estimates, its main limitation is a low number (up to 20,000) of contigs (contig splits) that can be clustered for human-guided binning. Visualization of larger datasets decreases the responsiveness of its interactive interface.

**Fig. 1:**
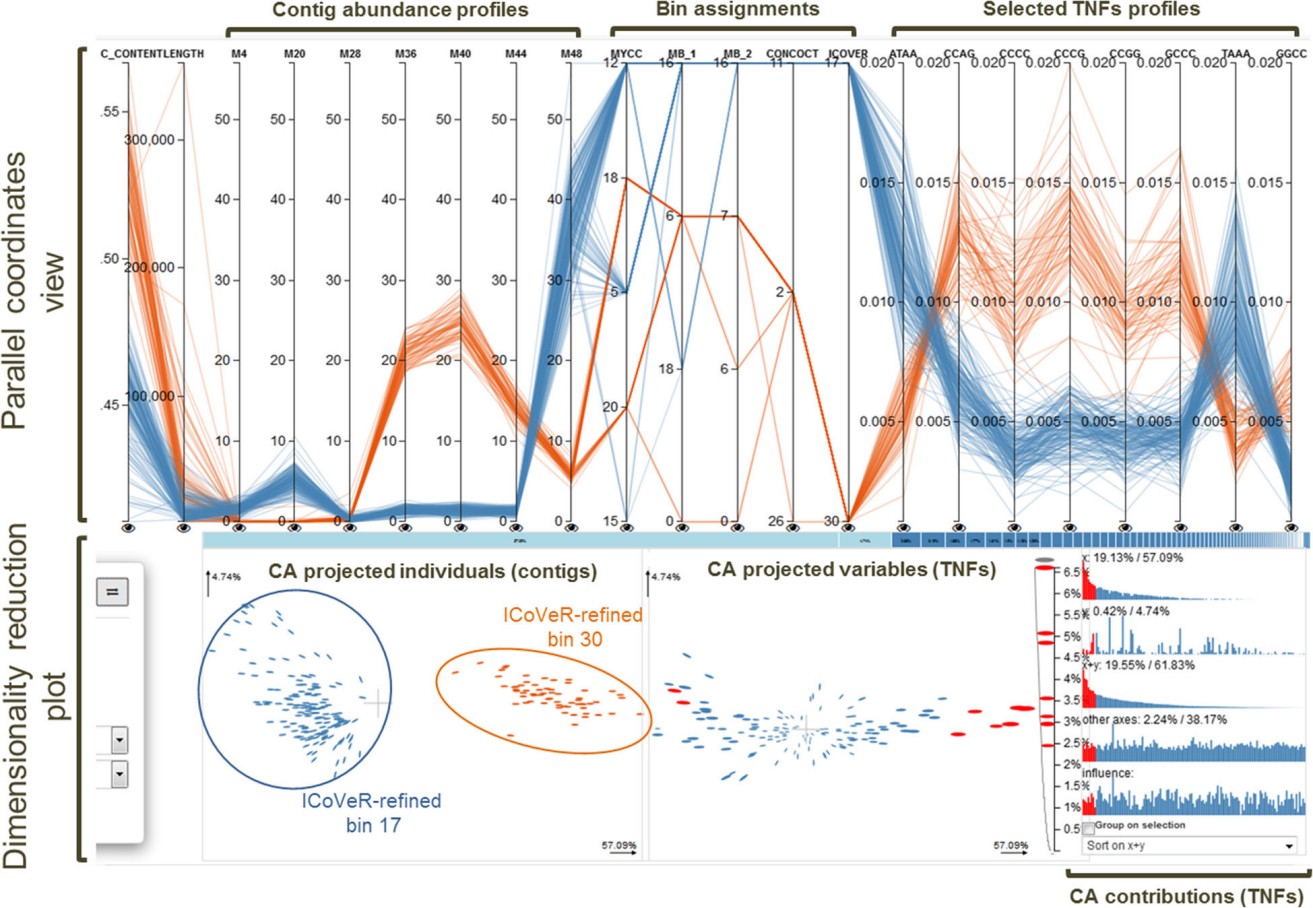
Static image from the ICoVeR interactive display of the AD microbiome dataset. ICoVeR-refined genome bins 17 and 30 are shown as examples

Here, we introduce ICoVeR, the Interactive Contig-bin Verification and Refinement visualization tool, which allows user-guided refinement of automatically generated contig bins. The software provides a visual interface that allows for comparing different binning results and their further supervised refinement. Its visualization window is composed of two connected and interactive main views: (1) a parallel coordinates view in which GC content, gene length, contig abundance spectra across different samples, binning results and TNFs values are displayed, and (2) a dimensionality reduction plot in which projections of the TNFs and contigs are shown (Fig. 1). To demonstrate ICoVeR’s utility we used it to refine automated binning results for a dataset representing the microbiome of an anaerobic digester (AD microbiome). We show that it improved the completeness and reduced the contamination of genome bins initially generated with MetaBAT [5], CONCOCT [3], and MyCC [6]. Moreover, we further used ICoVeR to refine genome bins for an infant gut metagenome [10], previously validated with CONCOCT [3], GroopM [4], MaxBin2 [11], MetaBAT [5] and MyCC [6].

## Implementation

### Overview of ICoVeR software

ICoVeR was designed as a visualization and refinement interface and not as primary binning software. It consists of data management and analytical functionality in the back end, and interactive visualization functionality in the front end. Data management and analytical functions of ICoVeR are implemented in R, thus the software is easily deployable on any system for which R is available. The front end of the application was developed using Web technologies. The interface between the R code and the web front end leverages OpenCPU [12], which provides an HTTP/REST API reflecting the functionality exported by the R package. The analytical functionality in the back end is added to drive the refinement process through the visual interface of ICoVeR. It is used to help focusing on relevant parts of the data, and to guide the decision making with respect to adding or removing contigs from a particular bin. Currently, it involves classic clustering and dimensionality reduction techniques, including the *k-means* [13] and correlation-based clustering. Correlation-based clustering is similar to the canopy clustering proposed by Nielsen et al. [14]. As opposed to the latter approach, the data items subject to clustering in our application are contigs rather than called genes. Here, the correlation clustering algorithm depends on two criteria: (1) the correlation threshold, which determines whether a contig is added to a bin with a given seed contig, and (2) the minimal cluster size, which determines the minimum number of contigs before a bin is considered a single cluster. The R Hmisc::rcorr function used for calculating the correlation-based clustering requires at least five variables (samples with calculated contigs abundances). In the case where a lower number of samples is available for the analysis, the user may choose to rely on the automated binning results only. Additionally, the user can extend the clustering of contigs abundances over samples to other variables, including the GC content or a selection of TNFs, or alternatively penta-nucleotide frequencies (PNFs). In addition to the clusterings, two dimensionality reduction techniques are provided, including correspondence analysis (CA) and principal component analysis (PCA; [15]). Both techniques are used to extract important information from a multivariate dataset in order to interactively select a subset of the potentially most interesting variables (samples or TNFs/PNFs) and individuals (contigs). The level of “interestingness” refers to variables (samples or TNFs/PNFs) that differ from the majority and thus may include patterns that may be important in driving differences between the individuals (contigs). While both PCA and CA result in orthogonal components, the former applies better to continuous numeric data (contigs abundance over samples), while the latter applies to categorical data (here TNFs and PNFs). As such, they represent straightforward methods for selecting a possibly interesting subset of variables to be visualized.

As a pre-requisite for ICoVeR, a user must provide: (1) a co-assembly file (in the FASTA format) containing all co-assembled contig sequences for all the metagenomic samples to be analyzed; (2) a contig coverage (abundance) file in the CSV format calculated for each metagenomic sample separately; (3) an ESCGs file in the CSV format containing all the predicted ESCGs for all co-assembled contigs; and (4) a clustering file in the CSV format (optional) with binning results from one or more automated binning tools that the user wants to refine. Although, the implemented *k-means* and correlation-based clusterings can perform an initial contig binning, it is highly recommended to start with dedicated automated binning software prior data visualization and refinement processes. A detailed installation guide is provided *online* https://git.list.lu/eScience/ICoVeR/wikis/home/. Once the package is installed it allows the software to be run in two different ways. Either the R OpenCPU package is used to start OpenCPU from an interactive R session, or a dedicated OpenCPU installation is used. The first option allows individual users to get started more quickly. The latter version scales better and allows for handling simultaneous requests.

### AD microbiome sequencing, co-assembly and data preparation

An anaerobic, mesophilic pilot-scale continuously stirred tank reactor (CSTR) of 100 L reaction capacity was inoculated and operated as previously described [16]. Seven samples (time points M4, M20, M28, M36, M40, M44 and M48) were selected for metagenomic analysis and the total genomic DNA was extracted with a PowerSoil DNA Isolation Kit (MoBio Laboratories Inc.), according to the manufacturer instructions. Sequencing libraries were prepared using a Nextera XTDNA Library Prep Kit (Illumina Inc.), and were pair-end sequenced (2 × 250 bp) in a single run on an Illumina MiSeq using a MiSeq Reagent Kit V2-500. Raw sequence data was deposited in the Sequence Read Archives (SRA) under the project accession number PRJNA303948. The resulting 26,371,696 metagenomic reads with average read length of 187.4 nt were imported to CLC Genomics Workbench v6.5 (CLC Bio), and trimmed using a phred quality score of 20 (limit of 0.01), minimum length of 50, and allowing no ambiguous nucleotides. A total number of 1,258,249 reads were completely removed after trimming. The seven metagenomes were assembled together using the CLC’s *de novo* assembly algorithm in mapping mode, using the following settings: word size of 48, automated bubble size, minimum contig length of 1000, mismatch cost of 2, insertion cost of 3, deletion cost of 3, length fraction of 0.9, and similarity fraction of 0.95. Assembly of over 20 million (M) reads (nearly 3.5 billion nt) led to 31,483 contigs (total length of 89,693,915 nt) with N50 of 3489 nt, and the longest contig of 416,944 nt. To determine the average coverage of contigs for each metagenome sample, reads were de-replicated and mapped back to the *de novo* assembled contigs using the RNA-seq analysis mode, with a minimum similarity of 0.8 over 0.9 of the read length, and using the ‘count paired-reads as two’ mode. In total, 73.02% of reads mapped back to the assembled contigs. The average abundance was calculated as DNA-RPKMs (here equal to the number of reads mapped to the contig and normalized by the contig length and per million mappable reads) and exported in BAM and CSV formats. TNFs and the GC content of the resulting contigs were calculated using functionality provided by the Biostrings package (detailed workflow is available at https://bioconductor.org/packages/release/bioc/html/Biostrings.html). Open reading frames (ORFs) on the resulting co-assembled contigs were first predicted with MetaProdigal [17], and subsequently ESCGs were identified as previously described [2, 18]. They are specific to the domain Bacteria.

### AD microbiome automated binning with MetaBAT, CONCOCT and MyCC

The AD microbiome dataset was initially binned using the fully automated MetaBAT, CONCOCT and MyCC binning software. To that purpose, respective BAM files were generated with CLC Genomics Workbench after mapping the quality trimmed reads to the resulting contigs, as described above. To generate genome bins MetaBAT was run using three different modes including ‘(very) sensitive’, ‘specific’ and ‘superspecific’. The lowest possible cut-off of 1500 for ‘minContig’ was used to valorize multiple short contigs. In addition the AD microbiome dataset was binned with CONCOCT using a cluster number set to 34. This number of clusters was chosen based on the estimated number of at least 30% complete draft genomes (based on the sets of 109 ESCGs) able to be recovered for the CSTR metagenome dataset. MyCC was run using the default parameters (except for using the ‘meta’ mode of Prodigal) and including the calculated contigs abundance information for the seven metagenomes. The binning results were further combined in a single CSV file and visualized with ICoVeR for further human-assisted bin refinement. Genome bins completeness and the level of contamination with foreign DNA were assessed with CheckM. An F1 score was used to weigh precision and recall of genome binning, and was calculated as previously described by [6]. Due to the lack of reference genomes, the CheckM calculated bin completeness was used as a recall value. The precision value was calculated as a difference of 100% minus the CheckM calculated bin contamination. For contamination values exceeding 100%, the precision value was set to zero. Paired-end connections for the different contigs grouping into the resulting genome bins were visualized with Circos [19], using the pipeline described in [2].

### Sharon’s dataset

A human infant gut microbiome [10] was used to further validate ICoVeR. The metagenome assembly along with the depth files (average contig’s abundance over 18 metagenomics samples) and binning information for CONCOCT [3], GroopM [4], MaxBin2 [11], MetaBAT [5] and MyCC [6] were downloaded from http://sourceforge.net/projects/sb2nhri/files/MyCC/Data/Sharon.zip and https://sourceforge.net/projects/sb2nhri/files/MyCC/Data/benchmark/Sharon.zip/download. These files were previously prepared as described in [6]. Only contigs above 1000 bp were considered (2329 contigs). The CheckM calculated bin completeness and contamination for CONCOCT, GroopM, MaxBin2, MetaBAT and MyCC bin assignements were taken from [6], (Additional file 1: Table S1 in that reference). For the ICoVeR-refined genome bins, bin completeness, contamination and F1 scores were calculated as explained above for the AD microbiome dataset.

## Results and Discussion

### ICoVeR implementation

ICoVeR provides an interactive visualization and refinement interface for the refinement of metagenomic contig bins resulting from multiple automated binning algorithms. Two clustering algorithms running in its back end work on rows that contain a variety of contig properties. While the widely used *k-means* clustering algorithm [13] is not suitable on its own to cluster full metagenomic datasets, we found it useful to enlarge or narrow down the selection of contigs during the refinement process. The correlation-based clustering is similar to the canopy concept previously proposed [14], however it allows the user to extend the contig coverages-based clustering to other variables such as GC content or a selection of TNFs, alternatively PNFs. This concept is similar to e.g. CONCOCT [3], which uses Gaussian mixture models to cluster contigs into population-level genomes based on sequence composition and coverage across multiple samples. Moreover, it allows a level of freedom that exceeds even the approach implemented in anvi’o [9], where the clustering profiles are currently limited either to coverage information, TNFs or the combination of both.

In addition to these two clustering algorithms, dimensionality reduction techniques are provided in ICoVeR for selecting variables and contigs of interest. CA is applied to TNFs (calculated by default) and PNFs, while PCA is useful to optionally select the most important samples (explaining the most variation retained by the dimensionality reduction technique) in case of large dataset composed of hundreds of samples, prior their visualization in the parallel coordinates view. Unlike other dimensionality reduction methods, e.g. modified t-SNE referred to as BH-SNE, initially implemented in VizBin [20] and more recently in MyCC [6], CA might not always capture the structure of the data in the most optimal way. However, by applying CA we can visualize not only the final projections but also the calculated contributions of the variables and individual contigs (the same refers to PCA, Fig. 1). These in turn allow for more informative views which provide a wide range of interaction opportunities to drive the refinement process.

The front end of ICoVeR is a Web application with two main views, including a parallel coordinates view [21] and a dimensionality reduction plot (Fig. 1). Both views are linked to each other and are highly interactive to support a *real-time* refinement process. The parallel coordinates view displays a vertical axis for each variable of the input dataset that is selected for visualization. Each contig is represented by a piecewise linear curve, which crosses each of these axes. The point at which a line crosses an axis represents the value of the corresponding contig for the variable represented by the axis. Lines in this view can be colored in different ways to ease the pattern discovery. By default, lines are colored based on GC content, but the user can choose any variable to color the lines by, or use a manual color mode where a color of choice can be assigned to a selection of contigs. The user can specify contigs of interest by making selections on one or several axes. Contigs which are selected are highlighted in the window. Next, the pane can be simplified by choosing either to keep the selected contigs or to remove them. The interactive CA dimensionality reduction plot shows the 2D projections of TNFs (default) and/or PNFs (optional) and the contigs. By default the threshold is set to display up to 5000 contigs in the dimensionality reduction plot. This plot focuses on refining individual clusters. As such this threshold reflects a trade-off between functionality and performance. Besides both projections, the dimensionality reduction plot shows additional extracted metrics to aid in assessing the importance of the projections as well as informing about the variables which are the most discriminative. By interacting with this widget, users can select variables of interest and look for structure in different projection planes. To help the interactive refinement process, CA is performed automatically on the TNFs data. This projection is updated automatically each time the contigs or bins are filtered. Selecting contigs in the contig projection view will highlight them in the parallel coordinates view and vice-versa. Selecting variables in the variable projection view will add them as axes to the parallel coordinates window. Thus, only variables capturing the structure which may render the refinement process more efficient can be selected. For an efficient visualization and refinement of more complex datasets, with a higher number of bins, a fish-eye zoom option was added to enable the selection of bins of interest from the parallel coordinates view [22]. Even on a modestly-sized desktop monitor (e.g. 30 x 38 cm) up to 50 bins can be easily handled without using the zoom.

Even though, most of the metagenomic datasets contain only a few samples, the accuracy of binning has been shown to improve as the number of samples (variables) increase [3]. Displaying too many samples in the parallel coordinates view without prior reduction can result in overcrowding and visual clutter. Automatically assessing the relative value of each variable could be used to rank these variables and present the user with the most informative ones only [23]. In ICoVeR, PCA can optionally be performed on all (selected) samples and the user can chose to display in the parallel coordinates view only the samples that explain the largest amounts of variation in the data (i.e. show the highest contribution). The same pre-selected samples can be further used for additional contig clustering.

The two main ICoVeR views are surrounded by additional features to support the verification and refinement process. There is a contig highlighter which allows highlighting a contig of interest. Furthermore, the interface provides a data counter which helps to keep a sense of how many contigs are in the current view. A tagging feature is provided to keep track of the refined bins. Finally, the ESCGs view shows in *real-time* the statistics for the current selection of contigs, based on the set of 109 ESCGs. It displays the information on how many of the expected genes are in the current selection as well as how many genes have two or more occurrences. This supports quick verification of completeness and contamination for the current selection of contigs.

### Automated bins assignment for AD microbiome

The AD microbiome dataset was selected to evaluate ICoVeR, since it represents a microbial (mostly bacteria and archaea) community of low to medium complexity that can be easily visualized and the generated genome bins can be easily refined in a reasonable period of time. Based on the previous results of the 16S rRNA gene amplicon sequencing, the CSTR metagenome was mainly comprised of members of *Bacteroidetes*, *Cloacimonetes* and *Firmicutes* [16]. The calculated indices of bacterial richness varied between 102 and 132 for the different samples. However, the 30 top dominant bacterial operational taxonomic units (OTUs) accounted for 80.54% ±4.53 of the whole bacterial community. Accordingly, using the determined number of 109 ESCG sets [18], the predicted number of at least 30% complete microbial genomes that should be recovered from this metagenome assembly was estimated at 34. Around 30 genomes should be at least 50% complete (according to the draft genome quality classification scheme proposed by [8]).

The AD metagenome was initially binned with MetaBAT, CONCOCT and MyCC, leading to disparate genome bins (Tables 1 and 2, Additional file 1: Table S1). Even though, these three different algorithms can combine sequence composition and contigs coverage information across multiple samples, the underlying algorithms are quite different. Nevertheless, our focus here was not on comparing the accuracy of the existing binning algorithms on the AD microbiome dataset, but on generating consensus-based genome bins from the disparate bins obtained with automated binning software and refined with ICoVeR. Binning with MetaBAT led to 34 bins assignments using the ‘sensitive’ or ‘specific' modes, and to 33 bins assignments using the ‘superspecific’ mode. Both ‘sensitive’ and ‘specific’ modes resulted in identical genome bins, while the ‘superspecific’ mode resulted in different bin assignments, which were in most cases more complete but contained a higher level of contamination (Tables 2 and 3). Regardless of the binning mode used, short contigs (below 2500 nt) were not incorporated to the bins, even when the limit was set to 1500 nucleotides. On the other hand, 34 CONCOCT-generated bin assignments were obtained. However, for the main part they were contaminated, as further determined by CheckM (Additional file 2: Table S2); most probably due to the loss of precision related to the low number of samples used for the AD microbiome assembly (seven samples). Indeed, the overall accuracy of CONCOCT starts to decrease below 50 samples [3]. MyCC has been recently identified as the most suitable binning and visualization tool when applied to small sample sizes [6]. Here, automated binning with MyCC resulted in 49 bins, 30 of which were around 50% complete microbial genomes with low (≤5%; 13 genome bins) and medium (5% to ≤10%; 10 genome bins) level of contamination (Additional file 1: Table S1).

**Table 1:** Summary of genome bins for AD microbiome dataset reconstructed with different binning algorithms

Binning algorithm	N^o^ genome bins	Completeness				Contamination			
		Near (≥90%)	Substantial (≥70 to 90%)	Moderate (≥50 to 70%)	Partial (<50%)	Low (≤5%)	Medium (5 to ≤10%)	High (10 to ≤15%)	Very high (>15%)
MetaBAT_1	34	8	7	5	14	31	3	0	0
MetaBAT_2	33	8	7	5	13	29	3	0	1
MyCC	49	12	8	10	19	29	14	2	4
CONCOCT	34	14	4	5	11	17	3	1	13
ICoVeR^a^	**31**	**11**	**8**	**10**	**2**	**30**	**1**	**0**	**0**

MetaBAT_1 - ‘sensitive/specific’ mode

MetaBAT_2 - ‘superspecific’ mode

^a^For ICoVeR (bold font), 31 pre-selected and the most complete genome bins (≥50% completeness based on MyCC genome bins) were refined

Completeness and contamination were calculated with CheckM. For CONCOCT the maximum number of clusters was setup to 34. The draft genome quality classification scheme is as proposed by [8]

**Table 2:** Completeness and contamination for 31 ICoVeR-refined genome bins for AD microbiome dataset

Bin^a^	Marker lineage^b^	Metagen. abund. %^c^	GC %	ICoVeR		MyCC		MetaBAT_1		MetaBAT_2		CONCOCT	
				Compl. %	Cont. %	Compl. %	Cont. %	Compl. %	Cont. %	Compl. %	Cont. %	Compl. %	Cont. %
1	*Bacteria* (UID2495)	22.3	36.1	**98.9**	**0**	98.9	0	98.9	0	98.9	0	100.0	18.8
2	*Bacteroidales* (UID2654)	6.3	41.7	**96.6**	**0.4**	98.9	39.2	96.6	0.4	96.6	2.2	100.0	82.3
3	*Clostridiales* (UID1212)	1.6	41.9	**95.3**	**1.3**	96.6	1.8	95.0	0.7	74.8	0.7	98.0	1.7
4	*Firmicutes* (UID241)	2.6	58.8	**94.9**	**2.0**	94.9	2.2	90.3	2.0	90.3	2.0	98.3	6.0
5	*Bacteria* (UID209)	1.5	42.0	**94.8**	**1.7**	94.8	3.7	90.8	0	90.8	0	94.8	2.0
6	*Euryarchaeota* (UID54)	1.5	59.9	**92.2**	**0.2**	94.7	4.7	85.8	0.1	85.8	0.1	94.6	42.8
7	*Bacteria* (UID2495)	1.1	51.9	**66.7**	**0.0**	94.7	51.0	66.7	0	66.7	0	100.0	101.7
8	*Bacteria* (UID2569)	8.8	46.7	**82.91**	**2.5**	93.4	23.8	75.6	7.7	75.7	9.8	100.0	99.4
9	*Bacteria* (UID209)	0.7	51.9	**92.8**	**3.2**	92.8	1.5	80.0	0.6	80.0	0.6	92.7	3.2
10	*Bacteria* (UID209)	1.0	51.1	**92.2**	**0**	91.4	1.7	93.6	6.8	93.6	6.8	98.2	19.1
11	*Bacteroidetes* (UID2605)	1.2	46.6	**85.9**	**3.8**	90.4	22.0	58.2	2.2	67.1	6.1	88.0	3.8
12	*Firmicutes* (UID239)	2.4	55.3	**89.8**	**2.7**	90.1	6.8	85.2	3.2	85.2	3.2	100	72.2
13	*Bacteria* (UID2495)	1.1	51.1	**87.3**	**2.2**	87.3	3.8	76.3	0	76.8	0	96.5	92.0
14	*Bacteria* (UID2495)	0.8	53.3	**83.8**	**0.6**	84.9	7.6	66.4	0.4	66.3	0.4	94.6	42.8
15	*Bacteria* (UID2570)	12.0	41.6	**81.6**	**2.5**	84.3	5.3	73.7	0.5	74.8	1.1	100.0	99.4
16	*Firmicutes* (UID1022)	0.7	54.2	**63.9**	**2.0**	83.4	17.2	48.0	0.2	43.8	0.2	100.0	72.2
17	*Bacteroidetes* (UID2605)	1.2	45.3	**91.2**	**0.6**	82.0	14.1	91.0	1.1	91.2	1.6	96.5	54.0
18	*Bacteria* (UID2495)	0.6	46.1	**76.3**	**3.8**	78.5	7.2	48.6	0	48.6	0	100	82.3
19	*Bacteria* (UID2982)	1.4	58.4	**77.3**	**5.3**	77.7	5.3	56.2	1.4	56.2	1.4	77.7	2.6
20	*Firmicutes* (UID241)	0.5	50.7	**60.4**	**2.1**	77.6	8.7	62.9	9.8	63.4	18.5	99.8	110.8
21	*Bacteria* (UID2565)	0.6	50.9	**65.5**	**1.1**	66.6	1.7	25.5	1.1	25.5	1.1	67.6	12.2
22	*Clostridiales* (UID1212)	0.3	45.4	**62.01**	**2.4**	63.7	4.0	NB		NB		63.0	5.1
23	*Thermoanaerobacterales* (UID1420)	0.6	35.8	**51.8**	**3.2**	63.7	7.3	28.9	0.1	29.0	0	51.8	3.2
24	*Bacteria* (UID209)	0.4	32.4	**66.8**	**3.6**	63.3	3.6	14.9	0	16.7	0	72.7	24.2
25	*Bacteroidetes* (UID2605)	0.5	49.9	**59.0**	**1.0**	60.0	5.0	46.2	0.7	46.2	0.7	75.5	3.01
26	*Bacteria* (UID2570)	0.3	49.6	**51.3**	**3.3**	56.8	10.2	NB		NB		96.6	54.0
27	*Firmicutes* (UID241)	0.3	52.3	**49.5**	**1.0**	56.8	6.0	12.8	0	NB		47.0	1.1
28	*Bacteria* (UID2569)	0.6	49.7	**54.5**	**2.1**	54.5	2.1	11.2	0	NB		62.4	15.3
29^d^	*Clostridiales* (UID1120)	0.1	47.4	**14.6**	**0.7**	53.7	8.1	NB		NB		45.2	3.8
30	*Bacteroidetes* (UID2605)	3.4	52.3	**99.5**	**0.5**	52.4	0.7	98.6	0.5	93.3	0.5	100.0	101.7
31	*Bacteria* (UID2495)	1.7	52.7	**95.6**	**0.1**	49.4	0	78.0	0.1	93.4	0.1	96.6	91.9

MetaBAT_1 - ‘sensitive/specific’ mode

MetaBAT_2 - ‘superspecific’ mode

^a^Number corresponds to the ICoVeR-refined bin (Table S2)

^b^Marker lineage was defined by CheckM

^c^Metagenomic abundance corresponds to the % of reads mapping to the contigs binned inside each bin

^d^Based on the wide range in the GC content, the pattern of contigs abundances across the multiple samples and the level of contamination, we judged this bin to be a mixture of several low abundant organisms. Therefore, the ICoVeR-refined genome bin was less complete than the corresponding MyCC and CONCOCT genome bins

Completeness and contamination were calculated with CheckM (highlighted in bold font for ICoVeR-refined bins). NB – no genome bin assigned

**Table 3:** Summary of binning performance for 31 ICoVeR-refined genome bins initially assigned with different binning algorithms for AD microbiome dataset

Binning algorithm	Average completeness (%)	Average contamination (%)	F1 (%)
MetaBAT_1	66.3	1.4	75.4
MetaBAT_2	70.4	2.2	79.3
MyCC	78.3	8.9	82.5
CONCOCT^a^	87.3	42.7	56.9
**ICoVeR**	**76.6**	**1.8**	**84.4**

^a^The relatively lower performance of CONCOCT on the AD microbiome dataset may be attributed to the loss of precision due to insufficient number of samples analysed (accuracy starts to decrease below 50 samples). Results for ICoVeR are highlighted in bold font

### Visualization and refinement of AD microbiome bins with ICoVeR

Although visualization with ICoVeR facilitates whole community analysis, for some datasets of high microbial complexity, the refinement process of all generated genome bins might be time-consuming. Here, we used CheckM to estimate the completeness and contamination of the resulting MetaBAT, CONCOCT and MyCC genome bins in order to prioritize most complete genome bins for further refinement with ICoVeR (Tables 2 and 3). In total, we selected 31 genome bins with around 50% of completeness, based on the CheckM results for the different automated binning algorithms (these bins can be visualised using a running instance of the tool preloaded with AD microbiome dataset; http://icover.list.lu:4200/ocpu/user/icover/library/ICoVeR/www/). A step-by-step description on how to load the data and refine contig bins is provided *online* for a few ICoVeR-refined genome bins (https://git.list.lu/eScience/ICoVeR/wikis/home/).

**Fig. 2:**
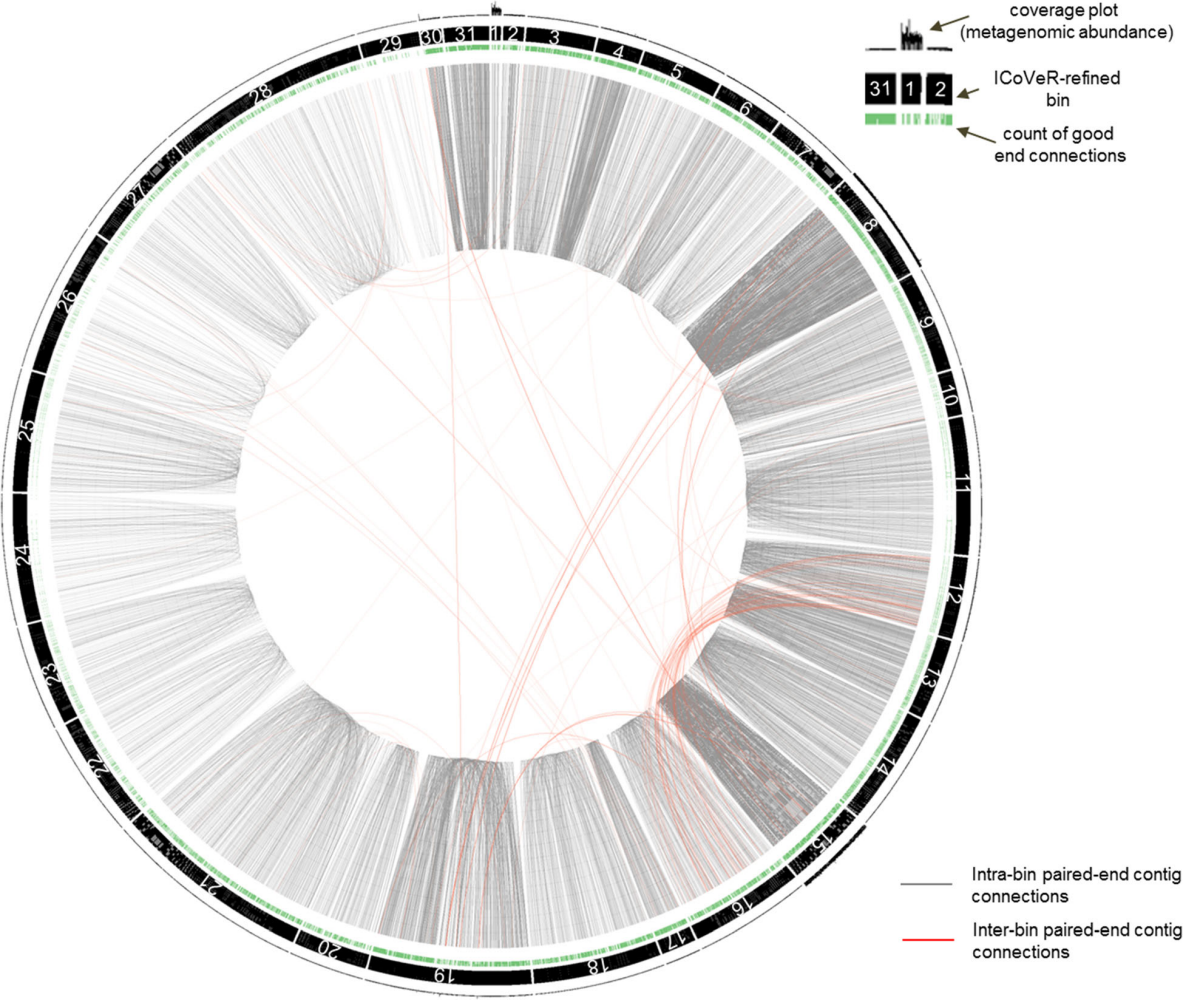
Visual representation of the paired-end connections for contigs grouping into the resulting genome bins for the AD microbiome dataset. Intra- (between the contigs inside the same bin) and inter-bin (between the contigs assigned to two different bins) paired-end contig connections are displayed as *grey* and *red lines*, respectively

None of the automated binning algorithms produced a single set of genome bins with highest completeness and lowest contamination (Table 2). Data visualization with ICoVeR helped to improve the completeness and to reduce the contamination for 23 out of 31 refined genome bins. Average completeness of ICoVeR-refined genome bins equaled 76.6% and contamination 1.8%. Even though the genome bins generated with CONCOCT and MyCC were in general more complete than the ICoVeR-refined bins, they both had higher levels of contamination (Table 3). Among the different binning algorithms, an average F1 score was the highest for ICoVeR-refined genome bins. Except for one genome bin, none of the ICoVeR-refined bins was characterized with the contamination exceeding 5%. Further tracking and visualization of pair-end reads mapping to assembled contigs, showed that out of nearly 15,000 good end connections, only 3.85% were between the contigs assigned to the different ICoVeR-refined genome bins (Fig. 2). This relatively low number of displayed inter-bin pair-end read connections confirms the overall low CheckM estimates of ICoVeR-refined bins contaminations (Table 2). On the other hand, this information could be integrated to the future release of ICoVeR to further improve the refinement process.

Metagenomic abundance of the refined genome bins was in a range from 22.3% (bin 1) to 0.1% (bin 29). Their size varied from 0.5 Mb (bin 29; 14.6% complete) to 3.37 Mb (bin 2, 96.6% complete). The GC content ranged from 35.8% (bin 23) to 59.9% (bin 6). Except for bin 6 that was assigned to *Euryarchaeota*, all other refined genome bins were of bacterial origin. Further biological interpretation of the obtained results is outside of the scope of the present paper and will be discussed elsewhere (manuscript in preparation).

### Refinement of genome bins with ICoVeR for a previously validated Sharon’s dataset

Recovering genome bins from mock communities (simulated datasets) was previously shown to yield very high precision (the absence of foreign DNA) and recall (percent of expected ESCGs that are binned) for the tested automated binning algorithms (compared to the same simulated datasets in [6]). Contigs derived from the same genome should ideally show similar abundance profiles across the different samples. In case of simulated datasets, the sequence coverage is nearly identical, what is reflected by 90–100% precision and recall of such contigs bins. However, due to the biases resulting from the available sequencing technologies, contigs coverages may vary quite significantly within and between the different samples in the case of real datasets [24, 25]. Additional biological factors e.g. recombination and/or horizontal gene transfer may render the accurate calculation of contigs abundances even more challenging. Therefore, to further demonstrate the effectiveness of ICoVeR we executed it on a previously validated Sharon’s dataset [10]. Genome bins of different completeness and contamination [6], formerly assigned by CONCOCT (32 genome bins), GroopM (13), MaxBin2 (10), MetaBAT (10) and MyCC (14), were visualized with ICoVeR (http://icover.list.lu:4200/ocpu/user/sharon/library/ICoVeR/www/). Based on the CheckM calculated completeness and contamination, we prioritized nine most complete bins for ICoVeR refinement. As a result, we obtained seven high-quality genome bins with completeness above 95% and contamination below 5% (strain heterogeneity was not taken into account; Table 4; Additional file 3: Table S3). By contrast, only five and four genome bins meeting the same criteria were generated with MaxBin2, MyCC and CONCOCT, MetaBAT, GroopM, respectively. Accordingly, the demonstrated advantage of combined bin refinement with ICoVeR against metagenomic binning results produced by separate binning tools was further supported by the superior average bin completeness, contamination and F1 scores (Table 5).

**Table 4:** Completeness and contamination for nine ICoVeR-refined genome bins for Sharon’s dataset

Bin^a^	Marker lineage^b^	ICoVeR		MaxBin2^c^		MyCC^c^		CONCOCT^c^		MetaBAT^c^		GroopM^c^	
		Compl. %	Cont. %	Compl. %	Cont. %	Compl. %	Cont. %	Compl. %	Cont. %	Compl. %	Cont. %	Compl. %	Cont. %
1	*Lactobacillales* (UID544)	**99.2**	**0**	99.2	0	99.2	0	100	204.2	99.2	0	99.2	0
2	*Clostridiales* (UID1120)	**98.9**	**0**	98.9	0	98.9	0	98.9	0	98.9	0	NB	
3	*Actinomycetales* (UID1530)	**97.9**	**0**	22.4	0	97.9	0	97.9	0	100	37.9	97.9	0
				66.9	0								
4	*Staphylococcus* (UID301)	**99.5**	**0.1**	99.5	0.1	99.5	0.1	99.5	2.9	99.5	2.9	99.5	0.1
5	*Staphylococcus* (UID294)	**95.9**	**0**	97	1.1	100	104.2	100	204.2	100	108.3	100	104.2
6	*Staphylococcus* (UID294)	**97.9**	**2.8**	97.9	3.4								
7	*Staphylococcus* (UID298)	**95.4**	**0.6**	100	24.4	95.4	0.6	95.4	0.6	95.4	0.6	95.8	1.7
8	*Staphylococcus* (UID298)	**84.1**	**0**	84.9	2.5	78.7	0	84.1	0	84.1	0	82.5	3.0
9	*Leuconostocaceae* (UID486)	**45.1**	**0.2**	72.8	37.9	45.1	0.2	45.1	0.2	37.7	0	24.7	0

^a^Number corresponds to the ICoVeR-refined bin (Table S3)

^b^Marker lineage was defined by CheckM. Completeness and contamination were calculated with CheckM (highlighted in bold font for ICoVeR-refined bins)

^c^Bin assignments for MaxBin2, MyCC, CONCOCT, MetaBAT and GroopM were downloaded from https://sourceforge.net/projects/sb2nhri/files/MyCC/Data/benchmark/Sharon.zip/download

**Table 5:** Summary of binning performance for nine ICoVeR-refined genome bins initially assigned with different binning algorithms for Sharon’s dataset

Binning algorithm	Average completeness (%)	Average contamination (%)	F1 (%)
MaxBin2	83.9	6.9	90.9
MyCC	89.3	13.1	71.7
CONCOCT	90.1	51.5	60.8
MetaBAT	89.4	18.7	68.6
GroopM	85.6	15.6	65.5
ICoVeR	**90.4**	**0.4**	**93.8**

Completeness and contamination were calculated with CheckM. Bin assignments for MaxBin2, MyCC, CONCOCT, MetaBAT and GroopM were downloaded from https://sourceforge.net/projects/sb2nhri/files/MyCC/Data/benchmark/Sharon.zip/download.. Results for ICoVeR are highlighted in bold font

### Current and future perspectives on genome bins visualization and refinement with ICoVeR

Even though most of the different automated binning algorithms that have emerged in the last couple of years combine both the TNFs alternatively PNFs information and the contigs co-abundances, their different underlying algorithms lead to disparate genome bins. Currently, there is no single, perfect tool for contig binning, however the binning variations could be further explored by refining the results of different binning tools. Visualization of multiple genome bins and further interactive refinement has been addressed previously by developing anvi’o [9], and currently ICoVeR. The principles behind the two tools are similar. Anvi’o in addition to its metagenomic workflow can perform further analysis of combined omics data, thus offering a dynamic work environment for comprehensive data exploitation. However, with respect to its application in refining the different automated binning results, the major drawback of anvi’o is the limited number of contigs (contig splits) that can be simultaneously displayed (currently up to 20,000) and refined. Here, we initially tested ICoVeR using a relatively small AD microbiome dataset (over 30,000 contigs), which was already too big to be entirely refined by anvi’o. A larger dataset (over 90,000 contigs; [26]) was also successfully displayed with ICoVeR and the complete set of files ready to be refined is available *online* for prospective users (https://git.list.lu/eScience/ICoVeR/wikis/seconddataset). Another advantage of ICoVeR in terms of big datasets is the very large number of samples (variables) that can be used for analysis, while only the samples capturing the contig co-abundance profiles which may render the refinement process more efficient can be selected to be displayed in the parallel coordinates view.

While ICoVeR currently allows users to include the output of different automated binning algorithms, one potential avenue for future work is to allow for algorithms such as CONCOCT, MyCC or MetaBAT to be configured and run as part of the ICoVeR workflow. However, currently this will require significant effort in terms of updating the application interface and architecture and would limit its usage to the Linux systems only. Thus, it would offer little benefit compared to including the results of these algorithms in the input data.

Quality of the recovered draft genome is crucial for determining its suitability for further analyses. Currently, the state-of-the-art estimation of the accuracy of generated genome bins relies on the presence or absence of ESCGs in contigs that form the specific bin [8]. In ICoVeR to reinforce the selection of contigs we rely on a selection of bacterial domain-specific 109 ESCGs [18]. Future implementation of a *real-time* display of the microbial lineage-specific information of completeness and contamination for a selection of contigs, would likely improve the quality of recovered genomes. Currently, this is determined by CheckM as a post-binning evaluation procedure [8]. In addition to ESCGs estimates, to facilitate the accuracy of the bin refinement process, information of linkage between contigs provided by the paired-end reads could be incorporated. This information has currently been explored in the recently published COCACOLA software (not tested in our study), where it was shown to improve the overall binning performance, especially when the number of samples is small [27].

The interactive visualization approaches allow the user to leverage their human-vision abilities to detect patterns, which a priori is subjective and may differ from one person to another. Even though the visualization-based metagenomic bin refinement process is supported by the ESCGs estimates, patterns of TNFs and/or contigs abundance spectra, *etc.*, it largely depends on a human-vision ability for pattern recognition. For this reason, it is difficult to accurately compare the outputs of the visualization-based tools using simulated or even previously published metagenomic datasets. Indeed, computational applications that are exploratory in nature, such as data visualization, involve many trial-and-error steps. Accurate reproduction of such analytical sessions would require the system to record the trail of user decisions and interactions in order to reproduce them at a later time. This concern about the provenance of insights and the reproducibility of findings using visual analytics tools has recently been an active research topic. Future use of an open source systems similar to e.g. VisTrails could capture the actual changes to data together with a detailed information on how these changes came about [28].

Additionally, to improve the visualization-based bin refinement process and to render it more reproducible, an algorithmic approach that combines bin predictions of several automatic binning tools into single, merged clustering should be envisaged. Recently, a method to merge suboptimal assemblies of the high-throughput sequencing reads was shown to successfully improve multiple assemblies of metagenomic samples, regardless of the data type, assembler used or parameter variation [7]. Such an automated approach applied to merge disparate genome bins would be highly interesting, and would potentially make full use of the available data for bin reconstruction, regardless of the algorithm settings.

## Conclusions

We present ICoVeR, a new interactive visualization interface for contig-bin verification and refinement. The software can visualize bin assignments from automated binning approaches, as well as perform further contig clustering using both contig co-abundances across multiple samples and their TNFs/PNFs features. ICoVeR has an open design that allows adding new algorithms and solutions that could further contribute to a better and faster genome bins refinement. We demonstrated the utility of ICoVeR by refining MyCC, MetaBAT and CONCOCT bin assignments for AD microbiome dataset. In addition, we applied ICoVeR to further refine genome bins for a previously validated Sharon’s dataset, formerly binned with CONCOCT, GroopM, MaxBin2, MetaBAT and MyCC. As a result, combining the strength of several binning algorithms led in many cases to nearly complete draft microbial genomes for both analyzed datasets. We also point to several improvements that would further render the bin refinement process faster and more replicable; such as an implementation of systematic mechanisms to capture the provenance of changes derived in the course of an exploratory task and an algorithmic approach to combine the different binning results into a single set of merged and improved genome bins before their visualization.

## Additional files

Additional file 1: Table S1. List of contigs and their bin assignments for the automated binning tools used in this study and for the subset of ICoVeR-refined genome bins for AD microbiome dataset. (XLS 2861 kb)

Additional file 2: Table S2. CheckM results for the genome bins generated with the automated binning tools used in this study and for the subset of ICoVeR-refined genome bins for AD microbiome dataset. (XLS 109 kb)

Additional file 3: Table S3. CheckM results for ICoVeR-refined genome bins for Sharon’s dataset. (XLS 56 kb)

## Abbreviations

AD: Anaerobic digestion
CA: Correspondence analysis
CSTR: Continuously/completely stirred tank reactor
ESCGs: Essential single copy genes
ORF: Open reading frame
OTU: Operational taxonomic unit
PCA: Principal coordinates analysis
PNFs: Penta-nucleotide frequencies
SRA: Sequence Read Archives
TNFs: Tetra-nucleotide frequencies
